# Correlations Between Immunophenotypic Markers and Clinical Progression in Romanian Patients Diagnosed with Diffuse Large B-Cell Lymphoma

**DOI:** 10.3390/medicina61060948

**Published:** 2025-05-22

**Authors:** Georgian Halcu, Anca Evsei-Seceleanu, Dana-Antonia Tapoi, Mihai Cerbu, Cristian Barta, Mihail Constantin Ceausu

**Affiliations:** 1 Catedra de Anatomie Patologică, Facultatea de Medicină Generală, Universitatea de Medicina si Farmacie Carol Davila Bucuresti, 050474 Bucharest, Romania; 2 Spitalul Clinic Coltea Bucuresti, 030167 Bucharest, Romania; 3 Spitalul Clinic Sfanta Maria Bucuresti, 011172 Bucharest, Romania; 4 Spitalul Universitar de Urgenta Bucuresti, 050098 Bucharest, Romania; 5 Institutul National de Endocrinologie C. I. Parhon Bucuresti, 011863 Bucharest, Romania

**Keywords:** diffuse large B-cell lymphoma, BCL2, immunohistochemistry, Romanian patients, lymphoma

## Abstract

Diffuse large B-cell lymphoma is a prevalent subtype of adult non-Hodgkin lymphoma; noted for its biological and clinical variability. *Background and Objectives*: This study seeks to assess the expression and prognostic implications of Ki-67, MYC, and BCL2 utilising immunohistochemistry on a cohort of Romanian patients diagnosed with DLBCL while also addressing the limitations imposed by the absence of fluorescence in situ hybridisation testing in resource-constrained settings. *Materials and Methods*: A single-centre, retrospective study involved 66 cases of formalin-fixed, paraffin-embedded tissue specimens obtained from patients with this lymphoma. *Results:* The median age at diagnosis was 61.81 years, with most individuals being 60 years or older; 59.1% of the patients were male. Our study identified that 65.2% of the cases belonged to the non-GCB subtype (ABC). MYC-positive expression was observed in 5 out of 66 cases (7.6%), and BCL2 protein expression exhibited a trend toward statistical significance, indicating a lower overall survival for BCL-2-positive patients. The expression of Ki-67 demonstrated a significant correlation with variations in overall survival (OS) (*p* < 0.001). Patients with low Ki-67 expression had an average survival duration of 76.39 months, contrasting with individuals exhibiting high Ki-67 expression, with a mean survival of 38.98 months. In conclusion, MYC, BCL2, and Ki-67 may be valuable prognostic indicator biomarkers. *Conclusions:* The prognostic significance of each biomarker varies based on the established cut-off point value. Future research should examine the relationship between protein biomarkers, morphological characteristics, and clinical outcomes in Romanian patients diagnosed with DLBCL, aiming to elucidate clinical ramifications and foster effective management.

## 1. Introduction

Diffuse large B-cell lymphoma (DLBCL) is among the most common adult non-Hodgkin lymphoma (NHL) types. Distinguished by considerable heterogeneity in its biological features and clinical course. It constitutes approximately 30–40% of all NHL cases [1,2].

In 2022, the fifth edition of the WHO Classification of Haematolymphoid Tumours (WHO-HAEM5) continued to endorse the molecular classification of DLBCL, maintaining the distinction between GCB and ABC subtypes, alongside the utilisation of immunohistochemistry as a pragmatic diagnostic methodology. Despite next-generation sequencing (NGS) advances revealing a complex mutational landscape in DLBCL, a comprehensive genetic model for integrating this information into the classification system remains absent. Notably, the classification of high-grade B-cell lymphoma characterised by dual rearrangements involving MYC and either BCL2 or BCL6 has been redefined as DLBCL with high-grade B-cell lymphoma with MYC/BCL2 (DLBCL/HGBL-MYC/BCL2). Cases exhibiting rearrangements of MYC and BCL6 are excluded from this categorisation. They are now classified as DLBCL, NOS, or high-grade B-cell lymphoma, NOS, contingent upon their morphological characteristics [2,3,4].

Clinically, at the time of diagnosis, the International Prognostic Index (IPI) may serve as a clinical tool for stratification; however, IPI fails to encompass the underlying biological diversity inherent in DLBCL.

Nevertheless, despite these advancements, molecular classification remains unavailable in most hospitals across Romania due to resource limitations. Consequently, IHC-based methodologies, such as Han’s algorithm [5,6], are widely utilised to classify DLBCL into GCB and non-GCB subtypes, employing three specific biomarkers: CD10, MUM1, and BCL6. Although this classification provides valuable insights for prognostic predictions, it still encompasses diverse patient subgroups, highlighting the underlying heterogeneity.

The WHO notes that there is currently no global consensus on which cases of DLBCL should undergo FISH. Routine FISH testing may result in increased diagnostic expenditures. Consequently, selective FISH testing—predicted by MYC expression and/or an elevated Ki-67 index, as determined by immunohistochemistry—is frequently recommended as a cost-effective screening strategy [7,8]. Furthermore, several studies have indicated no significant difference in overall survival rates between patients diagnosed with DHL and those with DEL, implying that these groups may exhibit similar biological behaviours [9].

The Romanian Society of Haematology guidelines recommend that in instances where FISH testing is not feasible, the expression of MYC and BCL2 proteins, as identified through immunohistochemistry, may suggest a worse prognosis compared to cases showing single or no expression [10]. Despite extensive research worldwide on biological markers in DLBCL [11,12,13,14], studies examining the clinical relevance and survival outcomes associated with protein expression identified by immunohistochemistry remain limited in the Romanian patient population [15].

This study aims to evaluate the expression and prognostic significance of MYC and BCL2 using immunohistochemistry on a group of Romanian patients diagnosed with DLBCL. It addresses the constraints imposed by the lack of FISH testing in resource-limited environments. Moreover, we want to acknowledge that several additional factors beyond diagnostic limitations influence prognosis in resource-constrained settings. These include restricted access to trained healthcare professionals, such as haematologists and oncologists; delays in diagnosis and treatment initiation; inadequate infection control measures; insufficient supportive care infrastructure; and the unavailability of newer, potentially more effective therapeutic agents.

Together, these barriers contribute significantly to treatment outcomes and must be considered when interpreting prognostic markers in such environments.

## 2. Material and Methods

### 2.1. Patients

**Scheme 1:**
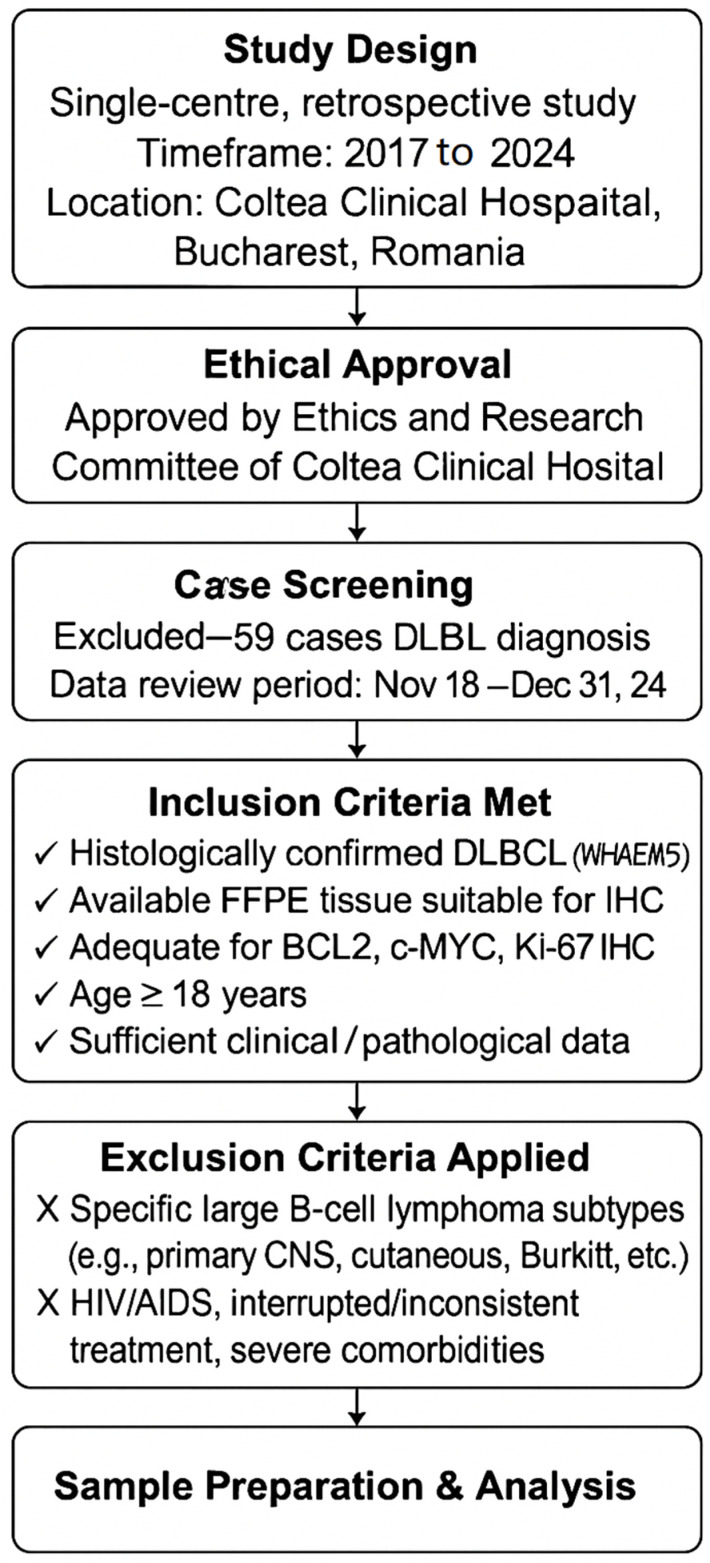
Flow diagram of materials and methods.

We designed a single-centre, retrospective study involving 66 cases of formalin-fixed, paraffin-embedded (FFPE) tissue specimens with DLBCL (Scheme 1). After authorisation from the Ethics and Research Committee of Coltea Clinical Hospital, patients aged 18 years or older who were diagnosed and treated at the Department of Haematology, Coltea Clinical Hospital, in Bucharest, Romania, were enrolled. Biopsy material (resection specimens or core needle) from patients diagnosed between 2017 and 2024 was meticulously reviewed by hematopathologists (GH, MC, and AES) to verify the diagnoses according to the WHO-HAEM5 guidelines.

Clinical data and DLBCL subtypes were retrieved from the electronic medical records from 18 November 2024 to 31 December 2024. This information was documented using a code system to maintain participant confidentiality and identities. There were initially 125 cases, but almost half of them lacked sufficient information in the hospital’s electronic system, or they chose to be treated elsewhere.

The inclusion criteria required a confirmed diagnosis:

- Patients must have a histologically confirmed diagnosis of DLBCL according to the WHO classification.
- Tissue availability: Available tissue from FFPE tumour samples suitable for immunohistochemical analysis.
- IHC Analysis: Adequate tumour sample for evaluation of BCL2, c-MYC, and Ki-67 protein expression by immunohistochemistry.
- Age: Adult patients aged 18 years or older at the time of diagnosis.
- Clinical Data Availability: Sufficient clinical and pathological data available, including treatment outcomes, staging, and survival information.

Exclusion criteria ruled out patients with specific large B-cell lymphomas, including primary CNS, cutaneous, intravascular, Burkitt, grey zone, plasmablastic, high-grade NOS, and primary mediastinal types. Individuals with HIV/AIDS who had inconsistent treatment for three years or significant treatment interruptions (defined as a consistent interruption or deviation from standard treatment protocols exceeding three months) were also excluded, along with those having concurrent solid tumours or severe comorbidities.

Paraffin blocks were sectioned into 2.5-micrometre-thick sections and stained with standard haematoxylin and eosin (H&E) utilising routine laboratory procedures. The histopathological features of these lesions were assessed and recorded in our database.

### 2.2. Microscopical and Immunohistochemical Examination

Sections with a thickness of 2.5 μm obtained from FFPE tissue samples were meticulously cut and affixed to positively charged microscope slides. Subsequently, these slides were subjected to a heating process at a temperature of 60 °C for one hour in a dry oven, thereby enhancing the adhesion of the tissue and softening the paraffin.

The immunohistochemistry procedure was conducted as per established protocol using a Ventana BenchMark ULTRA autostainer (Roche Diagnostics GmbH, Mannheim, Germany). The sections went through steps of deparaffinisation, rehydration, and antigen retrieval with Ventana’s CC1 solution (prediluted, pH 8.0, Roche Diagnostics GmbH, Mannheim, Germany). Primary antibodies were applied according to the manufacturer’s dilution guidelines and permitted to interact with the sections.

The primary antibodies utilised in this study included the following: anti-c-MYC (Y69) Rabbit Monoclonal Primary Antibody (clone Y69, Roche Diagnostic GmbH, Mannheim, Germany, ready-to-use), CONFIRM anti-bcl-2 (124) Mouse Monoclonal Primary Antibody (clone 124, Roche Diagnostic GmbH, Mannheim, Germany, ready-to-use), and CONFIRM anti-Ki-67 (30-9) Rabbit Monoclonal Primary Antibody (clone 30-9, Roche Diagnostics GmbH, Mannheim, Germany, ready-to-use). The incubation periods were established as 16 min for MYC and Ki67 and 32 min for BCL2. The visualisation process was conducted using the Ultraview universal DAB IHC detection kit (Roche Diagnostics GmbH, Mannheim, Germany), followed by counterstaining utilising haematoxylin and a bluing solution. The slides were meticulously cleaned and dehydrated through a graded series of ethanol and xylene. Finally, the slides were affixed with mounting media onto microscope slides.

MYC, BCL2, and Ki-67 slides were examined for each case at the highest magnification (40×) utilising the Aperio GT 450 DX Digital Pathology Slide Scanner (Leica Biosystems GmbH, Nußloch, Germany). The digital slides were evaluated employing Leica Aperio eSlide Manager/Leica Aperio ImageScope (Version 12.4.6.5003, Leica Biosystems GmbH, Nußloch, Germany). The positivity threshold was set at moderate to intense nuclear staining in ≥40% of cells for MYC and intense cytoplasmic staining in ≥70% of cells for BCL2. Any detected nuclear staining intensity with the Ki-67 antibody was classified as positive and contributed to the Ki-67 labelling index [12].

### 2.3. Statistical Analysis

All data from the study were analysed using IBM SPSS Statistics 25.0 and illustrated using Excel and Word (Version 16.97.1, Microsoft 365 Subscription 2025). Qualitative variables were written as counts or percentages and were tested between groups using Fisher’s Exact Test [16]. Quantitative variables were written as medians with interquartile ranges.

Kaplan-Meier analyses were used to evaluate the differences in overall survival between groups according to the analysed factors. According to the case, overall survival was estimated as means or medians with 95% confidence intervals. The 3-year survival rate and differences between groups were tested using the Log-Rank Test [17,18].

Additionally, Cox proportional hazard models were employed in both univariable and multivariable analyses to assess mortality risks [19]. Each variable’s effect was determined using hazard ratios accompanied by 95% confidence intervals and significance values. The models underwent evaluation for goodness of fit and significance levels, with α = 0.05 set as the threshold for all tests’ significance.

## 3. Results

### 3.1. Patient Characteristics

Our study involved sixty-six patients, with key clinical characteristics summarised in Table 1.

The median age at diagnosis was 61.81 years (IQR = 49.14–73.83), with most individuals being 60 years or older; 59.1% of patients were male. When revised, the Hans algorithm revealed that 65.2% of the cases were of the non-GCB subtype (ABC).

In the cohort, 7.6% had C-MYC immunohistochemical expression (5 cases), 57.6% were positive for BCL-2, and 65.2% were positive for BCL-6 (43 cases). Furthermore, 43 patients (65.2%) exhibited a Ki67 proliferation index of 75% or higher.

Regarding double and triple expressers, 7.6% of the cases exhibited double or triple expression. Specifically, one case (1.5%) had double expression of C-MYC and BCL-2, two cases (3%) were positive for C-MYC and BCL-6, and two cases (3%) were triple expressers (C-MYC, BCL-2, and BCL-6).

Table 2 shows no significant differences regarding age, gender, extranodal involvement, Ann Arbor stage, LDH value, or IPI risk score related to C-MYC, BCL-2, and Ki-67 expression (*p* > 0.05).

Germinal Center B-cell-like (GCB-like) subtype exhibited a significantly higher association with positive C-MYC cases, accounting for 80% compared to 31.1%. Conversely, the non-GCB subtype demonstrated a markedly stronger correlation with negative C-MYC cases, constituting 68.9% compared to 20% (*p* = 0.046). No significant associations were identified between the expressions of C-MYC, BCL-2, and Ki-67 (*p* > 0.05).

Other markers, such as MUM-1 (N = 43; 68.3%) and CD10 (N = 18 cases; 27.3%), did not show significant associations with Ki-67 expressions (*p* = 0.774 for MUM-1, *p* = 0.271 for CD-10).

However, for cell-of-origin subtypes, significant associations were identified for both biomarkers: MUM-1 with Non-GCB, showing 1 case (5%) of MUM-1 negative versus 40 cases (93%) of MUM-1 positive (*p* < 0.001), and CD10 with GCB, presenting 7 cases (14.6%) of CD-10 negative versus 16 cases (88.9%) of CD-10 positive (*p* < 0.001).

### 3.2. Survival Analysis

The median overall survival (OS) is 48 months (95% confidence interval = 26.22 to 69.77 months), with 36 recorded deaths representing 54.5% of the cases.

The disparities observed in overall survival contingent upon C-MYC expression (Negative: median = 48 months, 95% confidence interval: 23.9–72.1, 3-year overall survival = 53.1% vs. Positive: median = 60 months, 95% confidence interval: 0–134.87 months, 3-year overall survival = 60%, *p* = 0.353) were not statistically significant.

Upon evaluation of BCL-2 expression, patients exhibiting no expression demonstrate a median survival of 72 months (95% confidence interval: 36.56–107.43), accompanied by a three-year overall survival rate of 73.8%. Conversely, patients with positive BCL-2 expression have a median survival of 36 months (95% confidence interval: 26.2–45.8) and a three-year overall survival rate of 39.3% (*p* = 0.062). These findings suggest a trend of reduced overall survival in BCL-2 positive patients.

The expression of Ki-67 demonstrated a significant correlation with variations in overall survival (OS) (*p* < 0.001). Patients exhibiting low Ki-67 expression experienced an average survival duration of 76.39 months (95% CI: 62.07–90.72) and a three-year overall survival rate of 76.4%. Conversely, individuals presenting with high Ki-67 expression had a mean survival of 38.98 months (95% CI, 28.71–48.08) and a three-year overall survival rate of 40.7%. Elevated Ki-67 expression was associated with substantially decreased survival rates.

**Figure 1:**
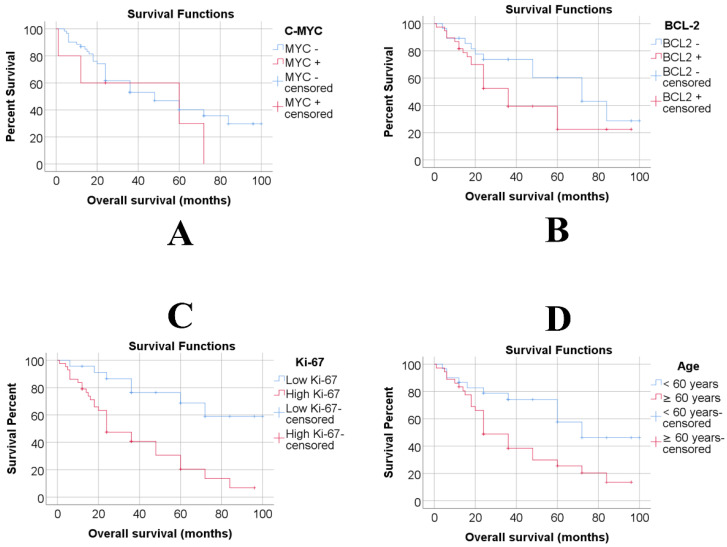
Overall survival of C-MYC (**A**), BCL-2 (**B**), Ki-67 (**C**) and Age (**D**) groups according to Kaplan-Meier analyses.

Additionally, individuals aged 60 years and older demonstrated a significantly lower survival rate compared to those younger than 60 years (younger than 60: mean = 68.51 months, 95% C.I.: 53.71–83.31, 3-year overall survival (OS) = 74.2% versus 60 years and older: mean = 41.14 months, 95% C.I.: 30.35–51.94 months, 3-year OS = 38.4%, *p* = 0.010), as illustrated in Figure 1.

The International Prognostic Index (IPI) risk score did not appear to significantly impact overall survival (OS) (*p* = 0.080).

The existence of double or triple expression did not significantly influence overall survival (OS) (*p* = 0.353). Furthermore, C-MYC/BCL-2/BCL-6 expression did not have a notable impact (*p* = 0.325), nor did C-MYC/BCL-6 expression (*p* = 0.636).

**Figure 2:**
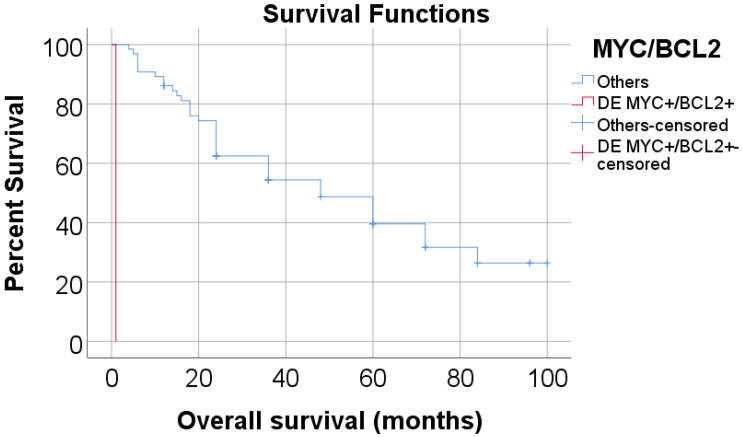
Overall survival of double expression groups of C-MYC/BCL-2 according to Kaplan-Meier analysis. (DE = Double expression).

However, in the case of C-MYC/BCL-2 double expression, significant differences in OS were observed (no double expression: mean = 53.52 months, 95% C.I.: 43.91–63.14, 3-year OS = 54.4% vs. double expression C-MYC/BCL-2: mean = 1 month, 95% C.I.: 1-1 months, 3-year OS = 0%, *p* < 0.001), as seen in Figure 2, however, considering that only one patient had this type of double expression, it is important to note that the power of the analysis is not very relevant.

The data presented in Table 3 indicate that after evaluating all potential mortality prediction factors, only age and high Ki-67 expression emerged as significant and independent predictors (*p* < 0.05). Specifically, patients aged 60 years and above faced a 2.36-fold increase in death risk (95% C.I.: 1.13–4.92, *p* = 0.022), while those with high Ki-67 expression had a 3.89-fold increased risk of death (95% C.I.: 1.67–9.07, *p* = 0.002).

## 4. Discussion

Diffuse large B-cell lymphoma is one of the most common non-Hodgkin lymphomas. It is a heterogeneous and aggressive type with various clinical manifestations. Research has focused on the roles of MYC, BCL2, and Ki-67 proteins in lymphoma development. Our study investigates the correlation between protein expression and clinical features in Romanian patients with diffuse large B-cell lymphoma (DLBCL), utilising established cut-off points. Due to limitations, FISH results are excluded; therefore, protein expression is assessed only via IHC, which restricts classification to the WHO-HAEM5. Nonetheless, these results offer significant prognostic insights, especially in resource-limited countries.

In our study, we found a correlation between protein expression and clinical outcomes. Diffuse large B-cell lymphoma primarily affects older adults, with a median age of 61.83 years (IQR 49.14–73.83) and a notable male predominance.

MYC-positive expression was found in 5 of 66 cases (7.6%) at a 40% threshold for this marker. The GCB-like subtype showed a significantly higher association with positive C-MYC cases (80% vs. 31.1%). Conversely, the non-GCB subtype showed a stronger correlation with negative C-MYC cases (68.9% vs. 20%, *p* = 0.046). Surprisingly, we found no association between MYC expression and Ki-67 expression at a 75% cut-off, inconsistent with previous studies [20]. Additionally, we examined MYC expression and overall survival (c-MYC negative: median, 48 months; 95% CI, 23.9–72.1; 3-year OS, 53.1% vs. c-MYC positive: median, 60 months; 95% CI, 0–134.87 months; 3-year OS, 60%; *p* = 0.353), revealing no statistically significant difference.

The MYC protein is a transcription factor critical for regulating the cell cycle, including processes like cell proliferation, growth, DNA replication, and protein synthesis [21,22,23]. In one-third of DLBCL cases, we observe increased MYC protein expression, suggesting it might be a useful prognostic biomarker. Note that the positivity threshold for protein expression varies by study based on specific criteria [24].

Our data showed BCL2 protein expression approached statistical significance, indicating lower overall survival for BCL-2-positive patients. This affects three-year OS by a 50% or greater cut-off. Conversely, BCL2 overexpression was not linked to the non-GCB subtype. Iqbal and associates noted that differing findings on BCL2 expression’s predictive value in DLBCL may result from multiple factors [25]. The heterogeneity in DLBCL cases arises from varying GCB and ABC subtype proportions. Patient cohorts may also exhibit risk factors beyond molecular classification, affecting outcomes. Variations in immunohistochemical staining, along with the pathologist’s expertise and interpretative subjectivity, contribute to inconsistencies across studies.

BCL-2 is an anti-apoptotic protein that promotes cell survival, and its overexpression in DLBCL is often due to gene amplification or chromosomal translocation. Despite this, the clinical relevance of BCL-2 expression in DLBCL is unclear, as studies report inconsistent associations with patient outcomes. The prognostic impact of BCL-2 overexpression also varies between the GCB and ABC subtypes of DLBCL [14].

We analysed Ki-67 expression using different cut-off values and determined that a threshold over 75% effectively distinguished Ki-67 expression; 43 cases had a Ki-67 index of 75% or higher, which showed significant differences in overall survival (OS) (*p* < 0.001) (Low Ki-67: mean = 76.39 months, 95% C.I.: 62.07–90.72, 3-year OS = 76.4% vs High Ki-67: mean = 38.98 months, 95% C.I.: 28.71–48.08 months, 3-year OS = 40.7%). Patients with high Ki-67 expression had significantly lower survival rates.

Ki-67 is a nuclear marker indicating lymphoproliferation levels in non-Hodgkin lymphoma and other tumours. It assesses proliferation and may have prognostic value for different DLBCL subtypes. Despite efforts to connect its expression with biological markers and clinical outcomes, no conclusive findings have emerged [26]. The varying definitions of low and high Ki-67 expression can create inconsistencies in studies using this vital marker. One reason may be the different threshold values used to determine Ki-67 expression status.

In addition, immunohistochemistry for Ki-67, c-MYC, and BCL2 is recommended to identify DEL, which is associated with a relatively poor prognosis and may help reduce the need for routine cytogenetic testing by FISH. However, since approximately 80% of DHL cases also exhibit DEL (co-expression of MYC and BCL2 proteins), IHC alone is insufficient for definitive classification. Immunohistochemistry cannot detect gene rearrangements, such as translocations; therefore, FISH remains essential for cases expressing these markers to identify DHL according to the WHO-HAEM5 classification accurately. This distinction is clinically significant, as DHL is associated with an even worse prognosis and typically requires more aggressive treatment approaches. As noted by Swerdlow, DEL should not be considered synonymous with DHL, and while DEL is more common, occurring in a more significant proportion of DLBCL cases, only about 20% of DELs meet the criteria for DHL, which itself is present in approximately 19% to 34% of cases [27].

## 5. Conclusions

In DLBCL patients, high Ki-67 expression (≥75%) was significantly associated with poorer overall survival, confirming its prognostic relevance. MYC and BCL2 expression showed subtype associations and trends toward worse outcomes, though statistical significance was limited. A single DEL case demonstrated an extremely poor prognosis. Immunohistochemistry offers a practical tool for prognostic assessment in resource-limited settings, though it cannot replace FISH for definitive WHO-HAEM5 classification.

## Figures and Tables

**Table 1:** Characteristics of the analysed patients.

Parameter	Value
Age (median (IQR))	61.81 (49.14–73.83)
Age ≥ 60 years (Nr., %)	36 (54.5%)
Sex (Male) (Nr., %)	39 (59.1%)
Cell of origin (Non-GCB) (Nr., %)	43 (65.2%)
Extra nodal involvement (Nr., %)	26 (39.4%)
Ann-Arbor stage (III-IV) (Nr., %)	55 (83.3%)
High LDH (Nr., %)	30 (45.5%)
IPI high risk score (>3) (Nr., %)	43 (65.2%)
C-MYC expression (Nr., %)	5 (7.6%)
BCL-2 expression (Nr., %)	38 (57.6%)
BCL-6 expression (Nr., %)	43 (65.2%)
Ki-67 ≥ 75% expression (Nr., %)	43 (65.2%)
Double/Triple expression (Nr., %)	5 (7.6%)
MYC/BCL2	1 (1.5%)
MYC/BCL6	2 (3%)
MYC/BCL2/BCL6	2 (3%)

IQR = Interquartile range, Nr. = Number of cases, GCB = Germinal Center B-Cell like, LDH = Lactate dehydrogenase, IPI = International Prognostic Index.

**Table 2:** Distribution of the patients according to marker expression and other characteristics.

Marker N, (%)	C-MYC		*p*	BCL-2		*p*	Ki-67 ≥ 75%		*p*
	Negative	Positive		Negative	Positive		Negative	Positive	
No.	61 (92.4)	5 (7.6)	-	28 (42.4)	38 (57.6)	-	23 (34.8)	43 (65.2)	-
Age									
<60	26 (42.6)	4 (80)	0.169	14 (50)	16 (42.1)	0.619	11 (47.8)	19 (44.2)	0.801
≥60	35 (57.4)	1 (20)		14 (50)	22 (57.9)		12 (52.2)	24 (55.8)	
Gender									
Female	25 (41)	2 (40)	1.000	11 (39.3)	16 (42.1)	1.000	8 (34.8)	19 (44.2)	0.601
Male	36 (59)	3 (60)		17 (60.7)	22 (57.9)		15 (65.2)	24 (55.8)	
COO									
GCB	19 (31.1)	4 (80)	0.046	11 (39.3)	12 (31.6)	0.604	10 (43.5)	13 (30.2)	0.295
Non-GCB	42 (68.9)	1 (20)		17 (60.7)	26 (68.4)		13 (56.5)	30 (69.8)	
Extranodal									
Nodal	38 (62.3)	2 (40)	0.375	18 (64.3)	22 (57.9)	0.621	16 (69.6)	24 (55.8)	0.304
≥1	23 (37.7)	3 (60)		10 (35.7)	16 (42.1)		7 (30.4)	19 (44.2)	
Ann-Arbor									
I-II	11 (18)	0 (0)	0.580	3 (10.7)	8 (21.1)	0.331	5 (21.7)	6 (14)	0.495
III-IV	50 (82)	5 (100)		25 (89.3)	30 (78.9)		18 (78.3)	37 (86)	
LDH									
Normal	34 (55.7)	2 (40)	0.652	15 (53.6)	21 (55.3)	1.000	13 (56.5)	23 (53.5)	1.000
High	27 (44.3)	3 (60)		13 (46.4)	17 (44.7)		10 (43.5)	20 (46.5)	
IPI score									
0–3	21 (34.4)	2 (40)	1.000	7 (25)	16 (42.1)	0.195	7 (30.4)	16 (37.2)	0.787
>3	40 (65.6)	3 (60)		21 (75)	22 (57.9)		16 (69.6)	27 (62.8)	
MYC									
Negative	-	-	-	26 (92.9)	35 (92.1)	1.000	22 (95.7)	39 (90.7)	0.651
Positive	-	-		2 (7.1)	3 (7.9)		1 (4.3)	4 (9.3)	
BCL-2									
Negative	26 (42.6)	2 (40)	1.000	-	-	-	10 (43.5)	18 (41.9)	1.000
Positive	35 (57.4)	3 (60)		-	-		13 (56.5)	25 (58.1)	
Ki-67 ≥ 75%									
Negative	22 (36.1)	1 (20)	0.651	10 (35.7)	13 (34.2)	1.000	-	-	-
Positive	39 (63.9)	4 (80)		18 (64.3)	25 (65.8)		-	-	

N = Number of cases, COO = Cell of origin, GCB = Germinal Center B-Cell-like, LDH = Lactate dehydrogenase, IPI = International Prognostic Index.

**Table 3:** Univariable and multivariable Cox-proportional hazard models are used in the prediction of mortality.

Parameter	Univariable		Multivariable	
	HR (95% C.I.)	*p*	HR (95% C.I.)	*p*
Age ≥ 60 years	2.46 (1.18–5.12)	0.016	2.36 (1.13–4.92)	0.022
Sex (Male)	0.98 (0.50–1.93)	0.967	-	-
Non-GCB	0.91 (0.46–1.81)	0.796	-	-
Extranodal	1.85 (0.95–3.60)	0.070	-	-
Stage III–IV	0.94 (0.38–2.30)	0.902	-	-
High LDH	1.19 (0.62–2.30)	0.591	-	-
IPI > 3	0.55 (0.27–1.10)	0.095	-	-
C-MYC	1.60 (0.56–4.56)	0.376	-	-
BCL-2	1.88 (0.93–3.79)	0.077	-	-
BCL-6	0.68 (0.34–1.35)	0.683	-	-
High Ki-67	3.98 (1.72–9.21)	0.001	3.89 (1.67–9.07)	0.002
Double/Triple Expressers	1.60 (0.56–4.56)	0.376	-	-

HR = Hazard ratio, C.I. = Confidence interval, GCB = Germinal Center B-Cell, LDH = Lactate dehydrogenase, IPI = International Prognostic.

## Data Availability

The raw data supporting the conclusions of this article will be made available by the authors on request.
